# Meetings of Societies

**Published:** 1927

**Authors:** 


### Meetings of Societies.

### Bristol Medico-Chirurgical Society.

THE Annual Meeting was held in the Society's room at the University on October 12th, 1927, the President and sixty-six members and guests being present. Mr. A. L. Flemming resigned the chair to his successor, Dr. W. K. Wills, who delivered his Presidential Address on "**Some Developments in Dermatology during the last Thirty Years.**"

### BRISTOL MEDICO-CHIRURGICAL SOCIETY.

### INCOME AND EXPENDITURE ACCOUNT for the Year ended 30th September, 1927.

### INCOME.

| | £ | s. | d. | £ | s. | d. |
|---|---|---|---|---|---|---|
| Members' Subscriptions .. | 238 | 16 | 6 | | | |
| Do., outstanding for 1926–1927 .. .. .. .. .. | 29 | 8 | 0 | | | |
| | | | | 268 | 4 | 6 |
| Interest on Bank Deposits .. .. | | | | 6 | 9 | 8 |
| | | | | £274 | 14 | 2 |

### EXPENDITURE.

| | £ | s. | d. | £ | s. | d. | £ | s. | d. |
|---|---|---|---|---|---|---|---|---|---|
| Grant to *The Bristol Medico-Chirurgical Journal* .. .. .. .. | | | | | | | 193 | 15 | 8 |
| Librarian—Honorarium .. .. .. | | | | | | | 15 | 0 | 0 |
| University Marshal .. .. .. .. | | | | | | | 1 | 0 | 0 |
| Miscellaneous Expenses— | | | | | | | | | |
| Cadena Cafe, Refreshments .. .. .. .. .. | | | | £5 | 7 | 0 | | | |
| Postages and Sundries.. | | | | 7 | 13 | 10 | | | |
| Printing and Stationery | | | | 34 | 1 | 6 | | | |
| Audit Fee .. .. .. .. | | | | 2 | 2 | 0 | | | |
| Lantern and Cinematograph at Meetings .. | | | | 3 | 3 | 0 | | | |
| Lewis's Medical and Scientific Library— | | | | | | | | | |
| Subscription | £4 | 4 | 0 | | | | | | |
| Postages .. | 0 | 6 | 1 | | | | | | |
| | | | | 4 | 10 | 1 | | | |
| | | | | | | | 56 | 17 | 5 |
| Balance, being excess of Income over Expenditure carried to next Account | | | | | | | 8 | 1 | 1 |
| | | | | | | | £274 | 14 | 2 |

### BALANCE SHEET as at 30th September, 1927.

### CAPITAL AND LIABILITIES.

| | £ | s. | d. | £ | s. | d. |
|---|---|---|---|---|---|---|
| Sundry Creditors, Audit Fee | | | | 2 | 2 | 0 |
| Capital as at 30th Sept., 1926 .. .. .. .. .. | 25 | 8 | 8 | | | |
| *Add* Arrears of Subscriptions paid up .. .. | 407 | 8 | 0 | | | |
| | 432 | 16 | 8 | | | |
| Balance of Income and Expenditure Account as above .. .. .. .. .. | 8 | 1 | 1 | | | |
| | | | | 440 | 17 | 9 |
| | | | | £442 | 19 | 9 |

### ASSETS.

| | £ | s. | d. |
|---|---|---|---|
| Cash at Bank—Current Account .. | 85 | 19 | 4 |
| Deposit Account .. | 327 | 12 | 5 |
| Members' Subscriptions for 1926–27 outstanding .. .. .. .. .. .. | 29 | 8 | 0 |
| | £442 | 19 | 9 |

Audited and found correct.

BRISTOL, 8th October, 1927.

H. E. KEELER, F.C.A.

The Secretary's report and the Treasurer's balance sheets were presented and accepted, and the following officers were elected :—

COMMITTEE.

*President*—Dr. W. Kenneth Wills.
*President-Elect*—Dr. H. L. Ormerod.

*Ex-officio.*

*Hon. Secretary*—Mr. W. A. Jackman.
*Hon. Medical Librarian*—Dr. W. A. Smith.
*Hon. Treasurer*—Mr. A. E. Iles.
*Editor of the Journal*—Dr. J. A. Nixon.

*Elected.*

Mr. F. Lace.

Mr. D. C. Rayner.

Mr. Hey Groves.

Mr. Duncan Wood.

Mr. T. Carwardine.

Mr. C. Ferrier Walters.

Dr. W. Vincent Wood.

Dr. R. S. S. Statham.

*University Library Sub-Committee.*

Dr. W. A. Smith. Dr. G. Parker. Mr. A. R. Short.

---

*November 9th*, 1927.

DR. W. KENNETH WILLS, President, in the Chair.

DR. NEWMAN NEILD read a paper on "Interlobar Empyema," in which he stated that neither interlobar empyema nor interlobar tuberculous effusion was particularly uncommon, although the diagnosis of interlobar empyema made by the physician might be apparently negatived at the operation. A frequent course of events was as follows :—

The first passage of the exploring needle through the compressed lung left a channel for the interlobar fluid to pass into the general pleural cavity. At this primary exploration the needle passed through lung before the fluid was reached, and before this there was no Grocco's triangle to be made out. A few hours later a Grocco's triangle was well marked, and a needle inserted at the site of the first exploration obtained fluid immediately within the parietal pleura, while if the needle went more deeply still, it passed through lung and again reached fluid. At the operation for empyema fluid was found at once on opening the parietal pleura.

Skiagraphy was of very great assistance, and he described the more usual appearances. The physical signs were practically confined to the lower lobe, the middle lobe being sometimes affected. There was gradually increasing impairment of resonance of this lobe, but an area behind and to the outer side of the angle of the scapula was much more dull than elsewhere. Whilst over the rest of the lobe breath sounds weakened until they disappeared, there was a sequence of impaired resonance, bronchial breathing, and absent breath sounds appearing from the centre of this dullest area, following each other centrifugally. At the outer limit of this area ægophony was often heard. The area ceased abruptly at the interlobar line in the axilla.

Dr. Neild illustrated his points by diagrams and detailed several cases upon which his conclusions were based. In these cases he preferred the usual operation for a general empyema to an operation at the point of greatest dullness in the axilla.

The difficulty of diagnosis based upon physical signs did not lie between interlobar and general empyema, but between interlobar empyema and "massive" pneumonia or occasional forms of pulmonary abscess.

He thought it probable that cases diagnosed as empyemata following pneumonias were frequently empyemata from the first.

The careful examination for this form of empyema would explain the cases, not uncommon amongst children, where an empyema was found, although breath sounds were still audible, though faintly, to the base. Also those empyemata that discharged through the bronchi or in the axillary region were probably interlobar forms.

He suggested that before a complete Estlander operation was performed a preliminary search should be made for a possible interlobar empyema that was preventing the expansion of the lung.

In opening the discussion on Dr. Neild's paper, the PRESIDENT alluded to the risk of hemorrhage following exploratory puncture of the chest wall.

MR. C. F. WALTERS thought that interlobar empyema often coincided with an ordinary empyema. When an interlobar empyema is explored operation should follow at once, and the needle track in the lung should be sterilised.

MR. C. A. MOORE made some comments on the surgical aspects of one of Dr. Neild's cases in support of his contentions.

DR. H. H. CARLETON said that he made it a rule not to puncture the pleura without local anæsthesia. He asked whether others could confirm his observation that where the pleural layers adhered to each other the sensibility of the parietal layer was lost.

DR. J. R. CHARLES thought that the persistence of a relatively quickened speed of respiration might suggest the presence of a metapneumonic interlobar empyema in patients who displayed no physical signs and were too ill for screen examination.

DR. NEILD replied.

